# Immune reconstitution inflammatory syndrome, with pulmonary and neurological cryptococcosis, in an HIV-negative patient

**DOI:** 10.1590/0100-3984.2015.0139

**Published:** 2016

**Authors:** Rodolfo Mendes Queiroz, Lara Zupelli Lauar, Marcus Vinicius Nascimento Valentin, Cecília Hissae Miyake, Lucas Giansante Abud

**Affiliations:** 1 Documenta - Hospital São Francisco, Ribeirão Preto, SP, Brazil.

Dear Editor,

A 26-year-old male presented with complaints of cough and fever for a few days. He reported having followed a weight loss program for the last four months, having lost 20 kg. He reported no comorbidities.

**Figure 1:**
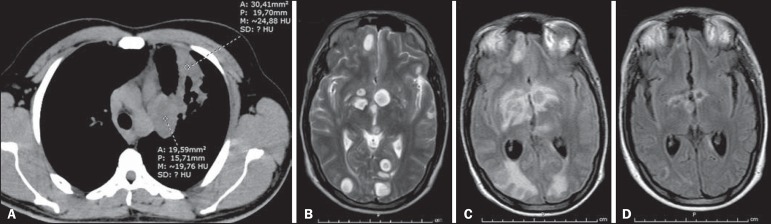
**A:** Axial computed tomography of the chest showing consolidation with air bronchogram and partially rounded hilar opacity, both presenting areas of low signal intensity, in the upper lobe of the left lung. **B:** Initial MRI of the brain. Scan with T2-weighted turbo spin-echo sequence showing multiple rounded cystic formations of varying dimensions scattered throughout the cerebral and cerebellar parenchyma, as well as in the left thalamus and nucleocapsular regions, having a discrete compressive effect and no signs of significant perilesional edema. **C:** Follow-up MRI of the brain. Scan with fluid-attenuated inversion recovery sequence, performed five weeks after the start of fluconazole therapy and the restoration of adequate nutrition, showing intense perilesional vasogenic edema indicating a reactive inflammatory process that was nearly undetectable at the beginning of treatment, due to immunosuppression. **D:** Follow-up MRI of the brain. Scan with fluid-attenuated inversion recovery-weighted sequence, performed five weeks after the start of corticosteroid therapy, when the patient was still under treatment with fluconazole, showing significant regression of the edema around the brain lesions, as well as a reduction in the size of the lesions.

Chest X-ray showed pulmonary consolidation in the left lung. A complete blood count showed leukocytosis and a lymphocyte count at the lower limit of normality. Subsequent X-rays, during antibiotic therapy, showed an increase in the consolidation. Computed tomography of the chest showed left lung consolidation with air bronchogram and a partially rounded hilar opacity, both containing areas of hypointense signals (Figure 1A), raising the hypothesis of an infectious or neoplastic process. Because he developed mental confusion, seizures, and postural instability, the patient was submitted to magnetic resonance imaging (MRI) of the brain, which showed multiple intraparenchymal cystic lesions (Figure 1B), with no enhancement and minimal edema at the borders.

Pathological examination of a biopsy sample obtained from the pulmonary consolidation revealed fungal infection with characteristics of deep cryptococcal mycosis. Staining with mucicarmine showed a mucin-positive capsule. The serology was negative for HIV, as well as for hepatitis B and C. Treatment was started with fluconazole, alternated with amphotericin B. During hospitalization, the general status of the patient became unstable and he was submitted to tracheostomy, subsequently developing tracheal candidiasis. After clinical improvement, he was discharged to outpatient follow-up, with home therapy and attention to an appropriate diet.

Five weeks after discharge, the patient was readmitted to the hospital with worsening neurological status. Another MRI of the brain showed the development of marked, progressive perilesional vasogenic edema (Figure 1C) and significant enhancement of the lesions by the paramagnetic contrast agent. Examination of the cerebrospinal fluid showed that there was no infection with *Cryptococcus* or any other agents, indicating an effective response to the treatment. The combination of clinical and radiological worsening, despite an effective treatment response, in a patient with evidence of immunosuppression (probably due to nutritional restriction), strongly suggested immune reconstitution inflammatory syndrome (IRIS). The antifungal therapy was maintained, and corticosteroid therapy was started, resulting in significant clinical improvement and regression of the lesions seen on follow-up imaging studies (Figure 1D).

In immunocompromised patients, cryptococcosis is common and is mainly caused by inhalation of *Cryptococcus neoformans* or *Cryptococcus gattii*^(1-4)^. Many patients with cryptococcosis develop pulmonary colonization, which is often asymptomatic. The symptoms become pronounced when they evolve to meningoencephalitis, presenting high tropism for the leptomeninges^(1-5)^. The presentations include masses and pulmonary consolidations^(1,3)^, gelatinous cystic formations in the brain, especially in the thalamus and basal nuclei^(2)^. The differential diagnoses include lung cancer with brain metastasis^(3,4)^. The diagnosis is made by identifying the fungus in sputum samples, bronchoalveolar lavage fluid, cerebrospinal fluid, histological sections, or culture^(4)^. Fluconazole and amphotericin-B are therapeutic options^(2,4)^.

Malnutrition is a known cause of immunodeficiency, affecting the mechanisms of adaptive immunity^(5,6)^. Some patients with acquired immunodeficiency syndrome or immunosuppression develop IRIS, in which the immune system begins to recover, responding to a previously acquired opportunistic infection with an exuberant inflammatory response that paradoxically causes a worsening of the symptoms^(2,7,8)^. Here, we have described an unusual case suggestive of cryptococcosis-related IRIS in an HIV-negative patient, probably developing immunosuppression due to nutritional restriction for weight loss.
